# On Lung Function and Interactions Using Genome-Wide Data

**DOI:** 10.1371/journal.pgen.1003174

**Published:** 2012-12-20

**Authors:** Erik Melén, Matteo Bottai

**Affiliations:** 1 Institute of Environmental Medicine and Centre for Allergy Research, Karolinska Institutet, Stockholm, Sweden; 2 Astrid Lindgren Children's Hospital, Karolinska University Hospital, Stockholm, Sweden; Georgia Institute of Technology, United States of America

Lung function reflects the physiological state of the lungs and airways, and abnormalities are present in asthma and chronic obstructive pulmonary disease (COPD), for example. Function measurements (e.g., forced expiratory volume in one second, FEV_1_) are often used in clinical practice to detect obstructive or restrictive conditions. Smoking is well-known to affect lung function negatively, presumably through induction of oxidative stress, inflammation, and lung damage. Studies also report negative effects on lung function and later asthma risk in children whose mothers smoked during pregnancy [1], which supports the “Barker hypothesis” that cardiovascular and respiratory diseases in adulthood may have their origin during fetal life [2]. Interestingly, transgenerational pulmonary effects of nicotine exposure during pregnancy, possibly mediated by epigenetic mechanisms, have recently been observed in animal studies [3]. Twin studies suggest a substantial genetic contribution to the variability of lung function, and several important loci have been identified in recent genome-wide association studies (GWASs) [4]. In addition, genetic factors of relevance for respiratory diseases are also proposed to influence lung growth in utero [5].

Although smoking is the most well-studied and established lifestyle risk factor for respiratory diseases, not all smokers develop diseases such as asthma or COPD [6]. Large individual variability in responses to environmental factors exists, and genetic susceptibility may partly account for this. For example, *IL13* single nucleotide polymorphisms (SNPs) have been shown to modulate the adverse effects of long-term cigarette smoking on pulmonary function [7]. *MMP12*, a protease involved in tissue degradation, has been associated with lung function and risk of COPD, but only in high-risk populations such as smokers and asthmatics [8]. The lungs develop during fetal life and throughout childhood [9], which is likely why children have been reported to be more susceptible to hazardous airborne substances compared to adults [10]. Whether this has any relevance for the identification of gene–environment interactions in adults or children remains to be investigated.

**Figure 1:**
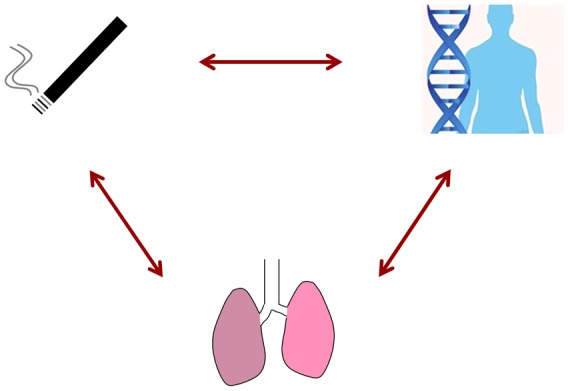
In this issue of *PLOS Genetics*, Hancock et al. address gene–environment interaction effects based on smoking status, GWAS data, and lung function outcomes in over 50,000 adults. This Perspective highlights the main findings in Hancock et al. [11] and discusses why interaction effects are so difficult to identify even in large, well-characterized data sets.

As the effects of gene-smoking interactions on lung function have not been extensively studied in large data sets so far, the paper by Hancock et al. in this issue of *PLOS Genetics* adds value to the current literature in many ways [11]. The authors present a large scale gene–environment interaction study based on GWAS data, smoking status, and lung function outcomes in over 50,000 adults from 19 studies primarily from the CHARGE and SpiroMeta consortia (Figure 1). Few similar studies on a complex trait where interaction effects have been thoroughly explored have been published to date.

Hancock et al. used a recently developed joint meta-analysis method (JMA) [12] primarily designed to detect genetic effects while taking environmental factors into account as the main method for their analyses. The method jointly tests main genetic (SNP) and interaction effects and is attractive for gene–environment interaction analyses thanks to its robustness and superior power over standard interaction models under certain conditions. Three novel regions of potential importance for lung function were identified in the present study: *DNER* (2q36), *HLA-DQ* (6p21), and *KCNJ2/SOX9* (17q24). Further, using the publicly available GEO repository, the authors were able to show differential expression of *DNER* and *SOX9* in epithelial cells from smokers versus non-smokers, which supports the involvement of these genes in smoking-induced lung function deterioration. In addition, recent experimental data from studies on mice show that tracheal and bronchial cartilage formation is regulated by *Tbx4* and *Tbx5* through *Sox9* expression [13]. Interestingly, *HLA-DQ* has been associated with asthma in independent GWASs of asthma [14], [15].

The most significant association was for rs7594321 in *DNER* with a *p*-value for the joint test equal to 5.0×10^−11^ in the pack-years model and 2.6×10^−9^ in the ever-smoking model. The corresponding interaction *p*-values were non-significant. In the ever-smoking model, stratified genetic effects of per allele change 0.049 in never-smokers (comparable to approximately a year and a half of FEV_1_ decline) and 0.035 in ever-smokers were observed. Thus, the effect of this particular variant seemed to only marginally differ between never-smokers and ever-smokers. In addition, standard interaction analyses identified no SNP by smoking interaction at the genome-wide significant level (*p*<5×10^−8^).

Using the JMA method, Hancock et al. successfully identified three novel loci not previously associated with lung function in GWAS or candidate gene studies. Yet, it is likely that important gene–environment interaction effects for lung function remain to be identified. Alternatively, one must accept that there are no gene-smoking interaction effects of importance for lung function after all. The latter alternative, albeit possible, seems to contrast with clinical and epidemiological evidence. Besides, clear and abundant evidence of interactions between gene and exposures has been found in animal studies and epigenetic projects [16], [17].

So why are interaction effects so difficult to identify even in large, well-characterized data sets? This question has not found a definite answer yet, and excellent reviews of methodological challenges and the current status of the research have recently been published [18], [19].

It has long been recognized that combinations of multiple variants of different genes may together raise the risk of complex diseases more than any single variant alone. Combinations of variants may in turn interact with one or more environmental factors. It is also sensible to allow for the possibility that any such combinations, if they exist, may not be identical for all individuals. The identification of possible combinations of variants, interacting environmental factors, and inter-individual heterogeneity presents major challenges that are best met by interdisciplinary efforts.

So far, most attempts to find interactions between individual or multiple gene variants and environmental factors at the genome-wide level have been undermined by insuperable limitations of statistical power and sample size. The recent advances in analytical methodology may have alleviated these limitations but surely not overcome them. The sheer number of gene variants in GWAS or genome sequencing studies, and the even greater number of their possible combinations, make it statistically unreasonable to pursue such quests with standard statistical hypothesis testing.

The wealth of information contained in the human genome, however, cannot be left untapped, and much can be done with the resources that are already available or expected to become available in the near future. Promising trails point to (1) development of exploratory analytical approaches that may help tackle high-order interactions between multiple variables; (2) acknowledgment and evaluation of the inter-individual heterogeneity observed clinically and experimentally through genomic, transcriptomic, and proteomic profiling; (3) investigation of the negative findings through power analyses that might help restrict the potential effect of some gene variants and rule out biologically or clinically relevant effects of many of them; and (4) critical assessment of the applicability to this context of the frequentist and Bayesian inferential paradigms and exploration of possible alternatives. Whatever trails are to be followed, research can only progress through close collaboration between disciplines like biostatistics, epidemiology, biology, and medicine.
